# Supplementary material for: Prevalence and socio-demographic correlates of depression and anxiety among late adolescents (15 to 21 years) in Mymensingh division, Bangladesh: A cross-sectional study
Source: PLoS One. 2025 Apr 29;20(4):e0320505. doi: 10.1371/journal.pone.0320505 (PMC12040267; doi:10.1371/journal.pone.0320505)
Supplement: Attachment — Submitted filename: Plos Comment for Author.docx [file pone.0320505.s001.docx]

**Comment for Author**

Thanks for receiving the opportunity to review your paper. Overall, I appreciate the thoroughness and importance of your study on **Prevalence of depression, anxiety and its association with socio-demographic factors among adolescents aged 15 to 21 in Mymensingh division, Bangladesh: Results from a cross-sectional study**. However, this manuscript must undergo minor revisions before it can be considered for publication.

The title needs to be modified; most of the research shows that adolescents are between 10-19 years or mention in title late adolescents age. The title should be **Prevalence of depression, anxiety, and its association with socio-demographic factors among late adolescents age in Mymensingh division, Bangladesh: A cross-sectional study.**

In abstract heading, introduction needs to change into background and keywords should be in MeSH form and alphabetical order.

**Introduction:** This introduction provides a good overview of the relevance of depression and anxiety among adolescents in Bangladesh. However, some references can be updated or expanded to reflect more recent studies, particularly those related to global mental health trends. The focus on Bangladesh is important, but perhaps elaborating more on the global context before zooming into Bangladesh could provide better framing

In introduction section **line 60-65 needs to verify According to the World Health Organization (WHO), depression and anxiety are the most common mental health disorders among late adolescents (15-24); where depressive disorders are characterized by sadness, loss of interest, feelings of guilt or low self-esteem, disturbed sleep or appetite, feelings of tiredness or poor concentration and anxiety disorders are characterized by feelings of fear, dread, and uneasiness (5).**With the provided reference, especially for the age group.

**Methodology:** The methodology section of the manuscript provides a detailed account of the study design and data collection process. However, several aspects need clarification and improvement to enhance the scientific rigor and reproducibility of this study.

The total number of 400 subjects is appropriate, but more detail is needed about sample size calculation.

Author need to mention how many researchers were involved in the data collection.

More information about potential biases in data collection, such as how respondents were recruited, would enhance transparency.

**Results:** Table 1 needs to cite in text.

**Discussion:** The discussion does a good job of comparing your findings to existing literature. However, the implications of your findings for future policy or practice could be expanded. What specific actions should be taken by policymakers or practitioners in Bangladesh as a result of your findings? Consider expanding on the limitations of the cross-sectional design in more detail. Additionally, potential avenues for future research based on your findings could be elaborated upon.

**Conclusion:** The conclusion is clear but could benefit from further emphasizing the practical applications of the study. For instance, what specific steps can be taken to improve mental health outcomes based on your findings?

**Comment to Editor:**

Overall, this manuscript makes a valuable contribution to the field of adolescent mental health, particularly in the context of an under researched population. The findings of this study offer important insights that can inform mental health policies and interventions in Bangladesh. The manuscript is well-structured, and although there are a few areas that could be improved, these are minor and can be addressed with minor revisions.
