# Supplementary material for: Prevalence and socio-demographic correlates of depression and anxiety among late adolescents (15 to 21 years) in Mymensingh division, Bangladesh: A cross-sectional study
Source: PLoS One. 2025 Apr 29;20(4):e0320505. doi: 10.1371/journal.pone.0320505 (PMC12040267; doi:10.1371/journal.pone.0320505)
Supplement: Attachment — Submitted filename: Response to Reviewers.docx [file pone.0320505.s002.docx]

**Response to Journals Requirements:**

| **SL** | **Reviewers’ comment** | **Author's response** |
| --- | --- | --- |
| 1 | Please ensure that your manuscript meets PLOS ONE's style requirements, including those for file naming. | Thank you for your comment we checked PLOS ONE’s style requirements and addressed them accordingly. |
| 2 | You indicated that you had ethical approval for your study. In your Methods section, please ensure you have also stated whether you obtained consent from parents or guardians of the minors included in the study or whether the research ethics committee or IRB specifically waived the need for their consent. | Thank you for raising this point. We unintentionally missed this point, but we obtained consent from the parents or guardians of the respondents aged below 18. We’ve now included this statement in the ethical consideration section under methodology. |
| 3 | We note that you have indicated that there are restrictions to data sharing for this study. For studies involving human research participant data or other sensitive data, we encourage authors to share de-identified or anonymized data. However, when data cannot be publicly shared for ethical reasons, we allow authors to make their data sets available upon request.  a) If there are ethical or legal restrictions on sharing a de-identified data set, please explain them in detail (e.g., data contain potentially identifying or sensitive patient information, data are owned by a third-party organization, etc.) and who has imposed them (e.g., a Research Ethics Committee or Institutional Review Board, etc.). Please also provide contact information for a data access committee, ethics committee, or other institutional body to which data requests may be sent.  b) If there are no restrictions, please upload the minimal anonymized data set necessary to replicate your study findings to a stable, public repository and provide us with the relevant URLs, DOIs, or accession numbers. Please see <http://www.bmj.com/content/340/bmj.c181.long> for guidelines on how to de-identify and prepare clinical data for publication. For a list of recommended repositories, please see <https://journals.plos.org/plosone/s/recommended-repositories>. You also have the option of uploading the data as Supporting Information files, but we would recommend depositing data directly to a data repository if possible.  Please update your Data Availability statement in the submission form accordingly. | Thank you for raising this issue. While obtaining ethical review and collecting data we didn’t obtain permission for data sharing from the respondents. However, for the betterment of the scientific community, we are okay to share data upon request. |
| 4 | We note you have included a table to which you do not refer in the text of your manuscript. Please ensure that you refer to Table 1 in your text; if accepted, production will need this reference to link the reader to the Table. | Thank you, we cited Table 1 in the text. |

**Response to reviewers’ comments:**

| **SL** | **Reviewers’ comment** | **Author's response** |
| --- | --- | --- |
| **Reviewer #1:** | | |
| 1 | Thanks for receiving the opportunity to review your paper. Overall, I appreciate the thoroughness and importance of your study on Prevalence of depression, anxiety and its association with socio-demographic factors among adolescents aged 15 to 21 in Mymensingh division, Bangladesh: Results from a cross-sectional study. However, this manuscript must undergo minor revisions before it can be considered for publication.  The title needs to be modified; most of the research shows that adolescents are between 10-19 years or mention in title late adolescents age. The title should be Prevalence of depression, anxiety, and its association with socio-demographic factors among late adolescents age in Mymensingh division, Bangladesh: A cross-sectional study. | Thank you for your valuable comment and guidance. We’ve made changes in the title now it read  *Prevalence and socio-demographic correlates of depression and anxiety among late adolescents (15 to 21 years) in Mymensingh division, Bangladesh: A cross-sectional study* |
| **2** | In abstract heading, introduction needs to change into background and keywords should be in MeSH form and alphabetical order. | We’ve addressed this comment and made changes accordingly. |
| 3 | Introduction: This introduction provides a good overview of the relevance of depression and anxiety among adolescents in Bangladesh. However, some references can be updated or expanded to reflect more recent studies, particularly those related to global mental health trends. The focus on Bangladesh is important, but perhaps elaborating more on the global context before zooming into Bangladesh could provide better framing In introduction section line 60-65 needs to verify According to the World Health Organization (WHO), depression and anxiety are the most common mental health disorders among late adolescents (15-24); where depressive disorders are characterized by sadness, loss of interest, feelings of guilt or low self-esteem, disturbed sleep or appetite, feelings of tiredness or poor concentration and anxiety disorders are characterized by feelings of fear, dread, and uneasiness (5).With the provided reference, especially for the age group. | Thank you for your valuable comment we have rewritten this section accordingly. |
| 4 | Methodology: The methodology section of the manuscript provides a detailed account of the study design and data collection process. However, several aspects need clarification and improvement to enhance the scientific rigor and reproducibility of this study.  The total number of 400 subjects is appropriate, but more detail is needed about sample size calculation. | Thank you for your valuable comment we have now included the sample size calculation in detail. |
| 5 | Author need to mention how many researchers were involved in the data collection. | Thank you. We have included this in the methodology section under the sub-heading “Study design, setting, and participants” |
| 6 | More information about potential biases in data collection, such as how respondents were recruited, would enhance transparency. | We have included this in the methodology section under the sub-heading “Study design, setting, and participants” |
| 7 | Results: Table 1 needs to cite in text. | Thank you for your comment we’ve cited this in the text. |
| 8 | Discussion: The discussion does a good job of comparing your findings to existing literature. However, the implications of your findings for future policy or practice could be expanded. What specific actions should be taken by policymakers or practitioners in Bangladesh as a result of your findings? Consider expanding on the limitations of the cross-sectional design in more detail. Additionally, potential avenues for future research based on your findings could be elaborated upon. | Thank you for bringing this up. We have added the implications of the findings for policy and practice in Bangladesh and some limitations in the discussion part. |
| 9 | Conclusion: The conclusion is clear but could benefit from further emphasizing the practical applications of the study. For instance, what specific steps can be taken to improve mental health outcomes based on your findings? | Thank you for your nice comment. We have now rewritten the conclusion to address your comment. |
| **Reviewer #2:** | | |
|  | Overall Comment: Thank you for your valuable work on researching adolescents' mental health issues. Your effort is commendable, and I appreciate the dedication that went into this study. However, I believe some revisions are required to translate it into a publishable paper as follows: | We appreciate your nice words. Thank you for your appreciation. |
| 1 | Introduction: It would be beneficial to include a paragraph that articulates your motivation for this study and outlines its novel and unique contributions. This will set a strong foundation for your readers. | Thank you for your valuable comment. We’ve outlined the novelty and unique contributions in the introduction. We couldn’t add the motivation for this study in a different paragraph, but we believe the introduction section has already addressed the motivation academically. |
| 2 | Methodology:   - In the subsection on study design, setting, and participants, you've mentioned the use of a multi-stage sampling technique. Please provide a justification for this method, along with relevant references. - You stated that Upazilas were randomly selected in the second stage, followed by unions and villages. However, the total number of Upazilas, unions, and villages and how many of them were chosen are not mentioned. Could you elaborate on the random selection process? Additionally, why did you limit your recruitment to rural adolescents, excluding urban and suburban populations? With potentially hundreds of thousands of adolescents available, how did you arrive at the 400 participants? Clarifying whether this was based on convenience or a first-come-first-served approach was adopted. | In response to your comment, we have revised this section and added the details procedures of the multi-stage sampling technique with its justification. We have included the total number of Upazilas and unions we’ve selected. For adolescent recruitment, we’ve recruited adolescents from both rural and urban areas of these selected upazilas. We also added the details sampling procedures in the methodology section. |
| 3 | Measures: The variables you've included require justification and references. Consider discussing why other relevant variables, such as the family history of depression or anxiety, educational outcomes, financial stress, household wealth, physical health indicators (e.g., BMI), drug addiction, frustration about life or love, etc. were not measured. | Thank you for your valuable comment we have included justification and references for used variables in the measure’s subsection of the methodology section. We’ve discussed the limitation of not including these variables and mentioned them in the conclusion part for further research implications. |
| **4** | Statistical Methods: Your paper currently lacks detail on the statistical methods used. Please include a discussion of ordinal logistic regression, highlighting its appropriateness for this study compared to other methods like OLS, multinomial logit, or probit, etc. | Thank you for your valuable comment. We have detailed the statistical methods including a discussion of ordinal logistic regression. We also compare other methods to highlight the appropriateness of using ordinal logistic regression. |
| **5** | Conceptual Framework: Instead of presenting your conceptual framework in the Statistical Analysis section, please create a dedicated section after the introduction. Moreover, Figure 1 indicates that socio-economic factors have both direct and indirect effects on depression and anxiety via lifestyle factors. If this is the case, you should illustrate how the indirect effects occur through your ordinal logistic regression using instrumental variables. If you wish to simplify the model due to insufficient instrumental variables in your dataset, consider modifying the figure to show only direct effects. | Thank you so much for your comment we’ve placed the conceptual framework after the introduction. We also develop a simplified model due to insufficient instrumental variables showing only direct effects. |
| **6** | Language Sensitivity: In your script, terms like “No education” and “father/mother had no education” may come off as imprudent. Consider using “without formal education” and father/mother without formal education” instead for a more respectful tone. | Thank you for your valuable observation. We’ve addressed these accordingly. |
| **7** | Results Section: On pages 11-12, you mention that “The ratio of moderate to severe depression was higher in (i) males vs. females, (ii) older adolescents (19-21 years) vs. years age groups, … , with severe anxiety (Table 2)” which are meaningless. Similarly, on pages 14-15, mentions that “The proportion of moderate to severe anxiety was higher in (i) males vs. females, (ii) older adolescents (19-21 years) vs. 15-16 years age groups, (iii) Muslim vs. non-Muslim…..” Please rewrite these findings in a more digestible manner. | Thank you for your nice comment. We have now revised the relevant sections to address your comment, and we believe it now reads better. |
| **8** | Specifications or robustness tests: Please include specifications or robustness tests for your ordinal logistic regression results to validate your findings. | Thank you for your suggestion. We have included robustness tests in the manuscript. |
| **9** | Discussion: Instead of solely comparing your findings to other studies, begin each paragraph in the discussion section by explaining your own results on relevant variables before making comparisons. | Thank you for your valuable comment. We’ve changed the discussion to address this comment. |
| **10** | Policy implications, limitations of the study, and suggestions for future research: Your study lacks discussion on policy implications, limitations of the study, and suggestions for future research. Add them in the conclusion section in detailed paragraphs. | We appreciate your comment and addressed these appropriately. |
|  |  |  |
| Reviewer #3: | | |
|  | **Overall Comment**  The authors have done commendable work in addressing a crucial and often under-researched issue of adolescent mental health in Bangladesh, providing valuable insights into the prevalence and socio-demographic factors associated with depression and anxiety. To further strengthen the manuscript, the following suggestions are offered: enhancing the clarity and structure of the abstract by including specific numerical findings and organizing it into subsections; refining the introduction to focus more on the local context and research gaps; providing a more detailed description of the sampling strategy, inclusion criteria, and validation of tools in the methods section; streamlining the presentation of results with a focus on significant findings, and incorporating stratified analyses; deepening the discussion with a critical comparison to existing literature and a more thorough exploration of implications for policy; and finally, including measures of effect size, interaction terms, and model fit diagnostics in the statistical analysis | Thank you for your insights, appreciation, and valuable comments. We have addressed your comments and the manuscript now reads better. |
| **1** | **Abstract** |  |
| **1.1** | The abstract lacks structure and detailed quantitative results. Although it mentions the prevalence of depression and anxiety, it does not provide sufficient breakdown of findings or statistical measures. | Thank you for your valuable comment, we have addressed this accordingly. |
| **1.2** | The objectives are stated generally, and the results do not clearly specify the key predictors or effect sizes. | Thank you for your valuable comment, we have addressed this accordingly. |
| **1.3** | Structure: Use subheadings like "Background," "Methods," "Results," and "Conclusions" for better clarity. | Thank you for your recommendations, we’ve changed accordingly. |
| **1.4** | Be Specific About Prevalence: Instead of saying, “Almost half (47.8%) of adolescents experience depression and over a third (32.8%) experience anxiety,” specify the severity levels, e.g., "47.8% had depression (---% moderate, ---% moderately severe, ---% severe), and 32.8% had anxiety (---% moderate, ---% severe)." | Thank you for your nice comment. We have now revised the relevant sections to address your comment, and we believe it now reads better. This now reads-  *The overall prevalence of depression was 47.8% (33.5% moderate, 10.5% moderately severe, and 3.8% severe depression), and anxiety 32.8% (31% moderate and 1.8% severe anxiety). Across most of the socio-demographic and lifestyle-related factors, the prevalence of depression and anxiety was higher among adolescent boys. Individuals aged 19-21 are most likely to experience depression and anxiety.* |
| **1.5** | Include Key Predictors: Mention the main predictors found in the regression analysis. For instance, "Depression was significantly associated with father's education level (p < 0.05) and lack of regular physical activity (p < 0.01)." | Thank you for your nice comment. We have rewritten the results section of the abstract. This now reads-  *The study found that unmarried adolescents, mobile phone users, rural adolescents, and those whose fathers and mothers were without formal education were more likely to experience moderate to severe depression and anxiety than their counterparts. The ordinal logistic regression revealed that adolescents whose fathers were without formal education were 2.77 times more likely to experience depression (95% CI = 1.21-6.35, p = 0.016), depression was more likely to be associated with those who were businessmen (OR = 1.95; 95% CI = 1.02-3.72, p = 0.043), and day laborers (OR = 0.41; 95% CI = 0.20-0.82, p = 0.012) and anxiety (OR = 0.05; 95% CI = 0.01-0.24, p < .000). Anxiety is prevalent among the adolescents without formal education (OR = 0.15; 95% CI = 0.03-0.75, p = 0.021), no family savings (OR = 0.42; 95% CI = 0.23-0.78, p = 0.006) and depression (OR = 0.06; 95% CI = 0.01-0.22, p < .000).* |
| **1.6** | Quantify the Associations: Report odds ratios (ORs) for key associations. For example, "Adolescents whose fathers had no education were --- times more likely to experience depression (95% CI: ---–---)." | Thank you for your valuable feedback, which helped us improve the abstract. We have rewritten the results section of the abstract. |
| **2** | **Introduction** |  |
| **2.1** | The introduction is broad and spends too much time on global mental health statistics without quickly narrowing down to the local context in Bangladesh. | We have made changes in the introduction section to address your comment. |
| **2.2** | The research objectives are somewhat buried and not explicitly detailed. | Thank you for your valuable feedback. We’ve rewritten the objective. This now reads-  *This study aims to (1) estimate the prevalence of depression and anxiety among adolescents aged 15-21 in Mymensingh, and (2) identify socio-demographic factors associated with these mental health conditions.".* |
| **2.3** | Focus on Local Context Early On: Begin by stating the lack of adolescent mental health research in Bangladesh, specifically in rural areas and among older adolescents (19-21). Use national statistics from the 2019 Bangladesh National Mental Health Survey early in the introduction. | We have made changes in the introduction section to address your comment. |
| **2.4** | Define the Research Gap Clearly: Emphasize the gaps that this study addresses, such as limited research on out-of-school adolescents or those living in rural areas. | Thank you for highlighting this issue. We’ve addressed this in the introduction. |
| **2.5** | Explicit Research Objectives: State the objectives clearly. For example: "This study aims to (1) estimate the prevalence of depression and anxiety among adolescents aged 15-21 in Mymensingh, and (2) identify socio-demographic factors associated with these mental health conditions, including parental education, physical activity, and rural versus urban residence." | Thank you for your valuable recommendation we’ve rewritten the objective accordingly. |
| **3** | **Methods** |  |
| **3.1** | The sampling process and criteria for inclusion/exclusion are not adequately detailed, making it difficult to assess the study's generalizability. | Thank you for your valuable comment we’ve made changes in the methodology section. |
| **3.2** | o There is insufficient explanation of the tools used (PHQ-9 and GAD-7) and how they were validated for use in the local context. | Thank you for your valuable comment, we have addressed this accordingly. This read-  *The PHQ-9 and GAD-7 were translated into Bangla for use in the Bangladeshi context. Previous studies in Bangladesh have shown these tools to be valid, with high internal consistency (Cronbach's alpha: PHQ-9 = 0.79-0.84; GAD-7 = 0.80-0.85) and robust psychometric properties across diverse populations, including adolescents (1). These validations confirm their appropriateness for diagnosing mental health conditions within the local cultural and socio-demographic context.* |
| **3.3** | Clarify Sampling Strategy: Expand on the multi-stage sampling technique. For example, describe the process for selecting districts, Upazilas, and villages, and how many participants were recruited from each stage. Specify if any steps were taken to ensure diversity in the sample. | Thank you for your valuable comment. We’ve rewritten the Study design, setting, and participants section to address your comment. |
| **3.4** | Explain Inclusion/Exclusion Criteria Clearly: Mention if adolescents with ongoing mental health treatments or specific conditions were excluded. Clarify how participants were approached, especially if they were out of school. | We’ve Mentioned the exclusion of the respondents with ongoing mental health treatments or specific conditions. This Reads  *Adolescents who were Currently receiving*  *treatment for mental health conditions or*  *had cognitive impairments that would not allow for accurate self-reporting were excluded from this study.*  The way participants were approached, especially if they were out of school also added at the end of the Study design, setting, and participants |
| **3.5** | Validation of Tools: Explain the reliability and validity of the PHQ-9 and GAD-7 scales in the local context. If these tools were used in other Bangladeshi studies, provide citations. Include any cultural adaptations made to the tools. | Thank you for your valuable comment. We’ve made changes to address this comment, focusing on the reliability and validity of the PHQ-9 and GAD-7 scales in the local context. We have also added citations of Bangladeshi studies using PHQ-9 and GAD-7. |
| **3.6** | Data Availability: Mention where the data can be accessed post-publication (according to PLOS ONE requirements) or explain the ethical restrictions. | Data availability is mentioned according to PLOS ONE requirements. |
| **4** | **Results** |  |
| **4.1** | The presentation of results is somewhat dense and difficult to follow, especially with large tables containing many variables. Non-significant variables are listed without further discussion. | Thank you for bringing this up. We’ve addressed your comment and placed only the significant variables in the table. |
| **4.2** | There is minimal stratification of results by key factors like gender, rural/urban residence, or age. | Thank you for your comment. Though we couldn’t discuss these stratifications due to the scope and space limitations, these are well articulated in all the analyses. |
| **4.3** | Streamline Tables: Focus on significant results in the main text and relegate non-significant findings to supplementary materials. For example, condense Table 2 by only presenting variables with p-values < 0.05 and showing their odds ratios. | Thank you for your comment we’ve changed Table 2 where variables with p-values < 0.05 are presented. |
| **4.4** | Include More Stratified Analyses: Break down the prevalence of depression and anxiety by gender and location (rural vs. urban). For example, report if males had higher depression rates in rural areas than females. | Thank you for your comment. Though we couldn’t discuss these stratifications due to the scope and space limitations, these are well articulated in all the analyses. |
| **4.5** | Graphical Representation: Add visual elements like bar charts or forest plots to make the results more accessible. For example, display odds ratios with confidence intervals for key predictors. | Thank you for your valuable comment. We’ve added visual elements in the manuscript to make the result more accessible. |
| **5** | **Discussion** |  |
| **5.1** | The discussion is more of a summary than a critical analysis. It does not adequately engage with existing literature to explain why some findings may differ from other studies. | Thank you for the comment. We’ve changed the discussion pattern and added a critical analysis of our findings. |
| **5.2** | There is minimal exploration of the implications of the findings for policy and practice in Bangladesh. | We have addressed this comment and increased the implications of the findings for policy and practice in Bangladesh in our write-up. |
| **5.3** | Engage Critically with Other Studies: Compare findings with previous studies in Bangladesh and nearby regions. For example, discuss why the current study found higher depression rates in males, while other studies reported higher rates in females. Suggest possible cultural, social, or methodological reasons for these differences. | Thank you for the comment. We’ve changed the discussion pattern and added a critical analysis of our findings. |
| **5.4** | Implications for Policy: Go beyond suggesting that mental health services need improvement. Provide specific recommendations, such as implementing school-based mental health programs or integrating mental health education in rural communities. | Thank you for your comment we’ve rewritten the recommendation and conclusion to address your comment. |
| **5.5** | Explain Limitations Thoroughly: The current limitations section is brief. Discuss potential biases (e.g., self-report bias), limitations due to the cross-sectional design, and any sampling issues that may affect generalizability. For example, mention the overrepresentation of rural adolescents and how it might skew the findings. | We appreciate your valuable thought. We’ve made changes to the recommendation to address your point. This is now read-  *This study has several limitations too. Firstly, this is a cross-sectional study which hampers causal attribution. The cross-sectional nature of the included studies only allowed an amalgamation of “associated factors” instead of ‘risk factors. Therefore, a longitudinal study is needed to clarify the causal relationship between depression and anxiety in adolescents and risk factors. Secondly, these results cannot generalize other divisions elsewhere in Bangladesh because the sample consisted of adolescents in one division. we could not completely rule out the possibility of recall bias. Our study data were collected based on self-report, which is prone to information bias.* |
| **5.6** | Future Research Suggestions: Recommend longitudinal studies or qualitative research to explore causality and understand the lived experiences of adolescents with mental health issues. | We have added future research suggestions accordingly. |
| **6** | **Conclusion** |  |
| **6.1** | The conclusion restates the findings without providing a strong summary of their implications or suggesting the next steps. | Thank you for your comment. We’ve rewritten the conclusion section accordingly. |
| **6.2** | Summarize Key Findings Clearly: For example, "This study found high prevalence rates of depression and anxiety, particularly among rural adolescents and those with low parental education, indicating an urgent need for targeted interventions." | Thank you for your comment. We’ve rewritten the conclusion section accordingly. |
| **6.3** | Convey Policy Recommendations Briefly: Instead of generic suggestions, be specific, e.g., "Policy efforts should focus on training healthcare workers in rural areas to identify and manage adolescent mental health issues." | Thank you for your valuable input. We’ve addressed it accordingly!!! |
| **6.4** | Avoid Repetition: Do not repeat points already discussed in detail in the results or discussion. Instead, provide a concise statement on the significance of the findings. | Thank you for your comment. We’ve rewritten the conclusion section accordingly. This now reads-  *In conclusion, this study found a high prevalence of depression and anxiety, particularly among rural adolescents and those who were students with low parental education, and highlighted several associated factors that were associated with depression and/or anxiety. Therefore, this research indicates an urgent need for targeted early interventions, prevention, and promoting mental well-being. Policy efforts should focus on parental involvement; training healthcare providers in rural areas, school-based mental health initiatives, and regulating social media to identify and manage adolescent mental health issues. Future research could uphold these findings by including more variable measures, such as the family history of depression or anxiety, physical health indicators (e.g. BMI), and frustration about life or love with longitudinal or qualitative studies among adolescents.* |
| **7** | **Tables and Figures** |  |
| **7.1** | Some tables are too detailed, making them difficult to interpret quickly. There is no use of figures to illustrate key findings. | Thank you for your comment. We’ve reduced the Table accordingly and added figures to illustrate key findings. |
| **7.2** | Condense Tables: For example, Table 2, highlight only significant results in the main manuscript. Move less relevant details to supplementary materials. | Thank you for your comment, Tables are condensed following your comment only significant results are presented. |
| **7.3** | Add Figures: Consider using a forest plot to illustrate the odds ratios and confidence intervals from the logistic regression. Add bar charts for the prevalence of depression and anxiety across different age groups or by gender. | Thank you for your valuable comment. The odds ratios seem better in the table. We’ve included a bar chart for the prevalence of depression. |
| **8** | **Statistical Analysis** |  |
| **8.1** | The analysis could benefit from including measures of effect size and considering interactions between key variables. | Thank you for your comment. We’ve Reported odds ratios (OR) with confidence intervals (CI) as measures of effect size. However, as interactions between key variables are not the hypothesized focus of this study, and keeping the scope and spaces in mind we drop it. |
| **8.2** | Include Effect Sizes (e.g., Cramér's V for Chi-Square Tests): This would add context to the significance tests. | Thank you for your suggestion to include effect sizes (e.g., Cramér's V for Chi-Square Tests). However, we chose not to include Cramér's V because of our limited sample size and distribution of variables. |
| **8.3** | Test the Proportional Odds Assumption: For ordinal logistic regression, confirm that this assumption holds. If not, consider using alternative models. | Thank you for your comment. We have tested the Model Fit Diagnostics using Pseudo R-squared. |
| **8.4** | Include Interaction Terms: Explore interactions, for example, between gender and rural residence, to see if the effects of socio-demographic factors on mental health outcomes differ across subgroups. | Thank you for your comment. Though we couldn’t discuss these stratifications due to the scope and space limitations, these are well articulated in all the analyses. |
| **8.5** | Model Fit Diagnostics: Report Pseudo R-squared values or other model fit statistics for the ordinal logistic regression. | Thank you for your valuable comment. We have reported pseudo-R-squared values in the manuscript. |
