# Supplementary material for: Prevalence and socio-demographic correlates of depression and anxiety among late adolescents (15 to 21 years) in Mymensingh division, Bangladesh: A cross-sectional study
Source: PLoS One. 2025 Apr 29;20(4):e0320505. doi: 10.1371/journal.pone.0320505 (PMC12040267; doi:10.1371/journal.pone.0320505)
Supplement: Attachment — Submitted filename: Response_to_Reviewers_auresp_2.docx [file pone.0320505.s003.docx]

**Response to Journal Requirements:**

| **SL** | **Reviewers’ comment** | **Author's response** |
| --- | --- | --- |
| 1 | Please review your reference list to ensure that it is complete and correct. If you have cited papers that have been retracted, please include the rationale for doing so in the manuscript text or remove these references and replace them with relevant current references. Any changes to the reference list should be mentioned in the rebuttal letter that accompanies your revised manuscript. If you need to cite a retracted article, indicate the article’s retracted status in the References list and also include a citation and full reference for the retraction notice. | I appreciate your input. we have thoroughly examined our list of references to make sure it is accurate and comprehensive. We’ve made substantial changes in the reference list of the manuscript. Our citations contained no retracted articles. |

**Response to reviewers’ comments:**

| **SL** | **Reviewers’ comment** | **Author's response** |
| --- | --- | --- |
| **Reviewer #1:** | | |
| 1 | Thank you for the opportunity to review your research paper. I wish you further success and look forward to seeing your work in print. | Thank you for your good wishes and for helping us improve the manuscript with your insightful feedback. |
| **Reviewer #2:** | | |
|  | Thank you for your revision. However, it is not yet adequate and complete. Please revise your manuscript further, addressing the following suggestions: | Thank you for your comment. We’ve carefully addressed all your comments. Hope this version will be okay to answer your queries. We’re thankful for your constructive comments and feedback. These really help to improve the quality of our manuscript. |
| 1 | In the abstract, you use different p-value specifications (e.g., OR = 0.15; 95% CI = 0.03-0.75, p = 0.021 or OR = 0.06; 95% CI = 0.01-0.22, p < 0.000). The p-value format should be consistent. Please use a uniform presentation, such as "<0.05" instead of "p = 0.021," throughout the abstract. | Thank you for your valuable comment and guidance. We have rewritten the abstract section following your comment. |
| 2 | I asked you to add a new section after the introduction for a Conceptual Framework and to discuss it in detail, explaining how each variable in the diagram relates to the prevalence of depression and anxiety. Instead, you added a brief summary. Please address this issue by providing a detailed, thoughtful discussion in 3-6 paragraphs in a separate Conceptual Framework section after introduction. | Thank you for your comment. We have addressed your comment and included a new section at the end of the introduction. |
| 3 | In the Methodology section, I requested an explanation of the random selection process of participants. However, you did not provide details on the methodology used, such as whether a lottery or random number generation was employed for random selection at each stage of sampling. Furthermore, I raised the question of how you choose 400 participants when there are potentially hundreds of thousands of adolescents available. There was no response regarding this crucial issue. Please explain in detail how the 400 participants were selected from the millions of adolescents in the study area, ideally in a separate paragraph. If randomly selected, which database you used and how did you access it, if not which other method. | Thank you for your detail comment. In our study, we randomly selected every unit but at the final stage due to lack of list or database of adolescents of the data collection area we had to select respondents conveniently following inclusion criteria. |
| 4 | I recommend that you justify and reference the variables you included in the study. “Please discuss why other relevant variables—such as family history of depression or anxiety, educational outcomes, financial stress, household wealth, physical health indicators (e.g., BMI), drug addiction, frustration about life or love, etc.—were not measured.” In your response, you stated that justification and references were provided in the methodology section. However, I found no such discussion in the revised manuscript. You need to clarify why the variables you selected were chosen and explain why others were excluded, citing relevant studies or references. | Thank you for your valuable comment. The justification for variable selection has now been specifically addressed in the Methodology section. The elimination of some variables due to issues with measurement, data dependability, and ethics has been supported by further references. In the Discussion section, we identify these shortcomings and recommend more research in these areas. This reads,  *Adolescent mental health is influenced by several factors, including a family history of depression or anxiety, educational outcomes, financial stress, household wealth, physical health indicators (e.g., BMI), drug addiction, frustration about life or love, etc. However, these factors were excluded for the following reasons:*  *Family history of depression or anxiety: Due to stigma and lack of official diagnoses, family histories of anxiety and depression are frequently underreported in Bangladesh (21,23,30). Furthermore, it was thought to be difficult for teenagers to accurately self-report on the mental health of their parents.*  *Educational outcomes: Although academic stress may play a role, our research concentrated on more general sociodemographic factors. Parental educational attainment was used to measure the indirect impact of education.*  *Financial stress, and wealth: We employed monthly family income, spending, and savings as a proxy for economic status in place of explicit financial stress measures, as these have been often employed in related research (12).*  *Physical health indicators (e.g., BMI): Physical health indicators e.g., BMI were excluded from data collection due to practical limitations in measuring anthropometric data in field-based surveys. However, we employed self-reported health satisfaction as a proxy following similar studies (19).*  *Drug addiction: We avoided difficult items that could result in underreporting because of ethical considerations and the self-reported nature of our study. Nonetheless, one relevant lifestyle component was smoking status.*  *Frustration about Life or Love: Although psychological stressors are significant, our study concentrated on behavioral and sociodemographic factors that have been validated in prior research and can be quantified objectively.* |
| 5 | Regarding statistical methods, your paper currently lacks detail on the methods used. You mentioned that ordinal logistic regression was discussed and compared with other methods to highlight its appropriateness for the study, but I could not find any detailed explanation of this in the revised manuscript. Please include a statistical methods section with a discussion of ordinal logistic regression, including its equation and parameters. | Thank you for your detailed feedback. We’ve made changes in the method section to address your comment. We have included a statistical methods section with a discussion of ordinal logistic regression, including its equation and parameters. The statistical analysis section is now read-  *Data entry, editing, and analysis were performed using the Statistical Package for the Social Sciences (SPSS) software program, version 26.0. We used descriptive statistics (e.g., frequencies, percentages, mean, standard deviation) and first-order analysis (i.e., chi-square tests). Variables that had a p-value of less than or equal to 0.05 were further analysed using ordinal logistic regression. Although multinomial logit and ordinal logistic regression are used for categorical data with more than two categories. The association of variables was considered statistically significant if the two-sided p-value was less than .05.*  *When the dependent variable of our study, depression and anxiety level, were is measured in ordinal scale (Minimal, mild, moderate, moderately severe, and severe) ordered categories so, we employed ordinal logistic regression (OLR). is best whereas when the dependent variable is categorical but has no link between the categories then should use multinomial logit. So, we think our data have ordered categories (Minimal, mild, moderate, moderately severe, and severe) and fit the ordinal logistic regression. OLR is suitable when the outcome variable is categorically ordered and allows us to estimate the probability of falling into a higher category of anxiety or depression while a few predictor variables are accounted.*  *Ordinal Logistic Regression Model:*  *Based on the cumulative odds technique, the ordinal logistic regression model makes the proportional odds assumption about the link between independent factors and the ordinal response variable. The following is a representation of the model:*  $Log(\frac{P\left( Y\leq j \right)}{P\left( Y>j \right)}= \beta0+\beta1X1+\beta2X2+\ldots+BkXk$  *Here,*  *P (Y≤j) is the cumulative probability of the outcome variable being at or below category j,*  *P(Y>j) is the probability of being above category j,*  *β0 is the intercept,*  *X1, X2, ..., Xk are the independent variables (e.g., age, gender, parental education, family income, physical activity, smoking),*  *β1, β2, ..., βk are the regression coefficients representing the change in the log-odds of being in a higher category for a one-unit increase in the predictor variable.* |
| 6 | In response to my comment 8, you mentioned that "robustness tests have been included in the manuscript." However, I could not find any robustness tests or results in the revised manuscript. Additionally, you have 23 variables in the study, but only 8-9 variables are included in the ordinal logistic regression, which suggests that your model may be manipulated. To avoid bias, you need to incorporate all explanatory variables mentioned in the descriptive statistics and run the ordinal logistic regression with these variables included. Also, variables such as age, gender, and family income are crucial. Why were they excluded from the regression? These variables need to be included, and the regression should be rerun to ensure accurate results. | Thank you for your comment, and we’re very sorry that we mistakenly forgot to add this. Now we’ve added robustness tests in methodology section of the manuscript. Following best practices in public health research, we first reduced over fitting and multicollinearity by including only variables with p < 0.05 from the bi variate analysis in the ordinal logistic regression (Jeelani et al., 2022; Magklara et al., 2015b; Mohamad et al., 2021). We do, however, recognize the worry about possible bias resulting from variable exclusion. The consistency of the results confirmed the strength of our conclusions. To improve interpretability and prevent statistical noise, we stuck with the stepwise strategy, making sure that only variables that had been shown to be significant in bi variate analysis were included in the regression. |
| 7 | In the discussion section, you included a lot of information from other research but failed to properly discuss your own findings. You need to first compare your findings with those from other studies and then explain how the local context of your study area may account for differences from another research. This comparison has not been done adequately. | Thank you for your comment. We have revised the discussion to address your comment. |
| 8 | In comment 10, I asked you to add a discussion on policy implications, study limitations, and suggestions for future research in the conclusion section. Unfortunately, no improvement has been made in this regard. Please rewrite the conclusion in 3-5 detailed paragraphs: one sentence on background and methodology, one sentence on results and findings, one on policy suggestions based on your findings, one on limitations, and one on future research. This should be done thoroughly. | We appreciate your thoughtful comments, and we have fully rewritten the conclusion section to address your comment. |
| 9 | You’re finding that "unmarried adolescents, mobile phone users, rural adolescents, and those whose fathers and mothers were without formal education were more likely to experience moderate to severe depression and anxiety than their counterparts" should be reflected in policy suggestions. In separate sentences, provide specific policies, programs, or actions that can address each of these variables and help improve mental health outcomes. | Thank you for your comment. We have address this in the policy recommendations. |
| **10** | In the abstract, you mentioned that "depression was more likely to be associated with those who were businessmen and day laborers." This should be analyzed in the results section. You need to explore why these groups are vulnerable to depression and suggest policies to reduce mental stress among these working groups. | Thank you for your insight. We have already analyzed businessmen and day labourer in the result section. Why these groups are vulnerable to depression are included in discussion part and policy suggestion for this groups have added accordingly. |
| 11 | The last paragraph you added in the discussion should be rewritten in clearer, more readable language and placed in the limitations paragraph of the conclusion. | Thank you and we have rewritten the whole conclusion part. |
| **12** | In my previous comments 7 and 8, I requested clarification and made suggestions to improve readability. However, the revised manuscript still contains sections that are not audience-friendly or easy to read. Please revise these paragraphs to improve clarity and coherence. | Thank you for your suggestions. We have addressed this comment and improve the manuscript to make it audience friendly. |
| **Reviewer #3:** | | |
| **1** | The manuscript effectively addresses a critical and under-researched issue of adolescent mental health in Bangladesh, providing valuable insights into the prevalence of depression and anxiety and their socio-demographic correlates. The authors have made significant revisions based on reviewer feedback, improving the structure, clarity, and scientific rigor of the study. Key strengths include the detailed statistical analysis with effect sizes, the inclusion of specific policy recommendations, and a critical comparison with existing literature. However, a few minor improvements can further enhance the manuscript's quality. These include explicitly clarifying whether the proportional odds assumption was tested and expanding on the implementation challenges of proposed policies in the Bangladeshi context. Overall, the manuscript is well-prepared and, with these refinements, is suitable for publication, contributing meaningfully to the global conversation on adolescent mental health. | Thank you for your nice comment. Your insightful comment helps us a lot to improve the overall quality of this manuscript. We have addressed this comment in methodology and conclusion section. We tested and shared the findings of the proportional odds assumption in updated manuscript. Besides we have also described the implementation challenges of proposed policies in the Bangladeshi context. Thank you for being optimistic and helping to improve the overall quality of this manuscript. |
